# Effects of herbal dregs supplementation of *Salvia miltiorrhiza* and *Isatidis Radix* residues improved production performance and gut microbiota abundance in late-phase laying hens

**DOI:** 10.3389/fvets.2024.1381226

**Published:** 2024-05-03

**Authors:** Zhaonian Li, Ning Ma, Xincheng Gong, Wanyu Shi, Xianhua Meng, Jingjiao Yan, Zhiqiang Zhao, Jiefeng Li

**Affiliations:** ^1^Institute of Animal Husbandry and Veterinary Medicine of Hebei Province, Baoding, China; ^2^College of Traditional Chinese Veterinary Medicine, Hebei Agricultural University, Baoding, China; ^3^Hebei General Station of Animal Husbandry, Shijiazhuang, China; ^4^Animal Husbandry Technology Promotion Institution of Zhangjiakou, Zhangjiakou, China

**Keywords:** Chinese medicine residue, hens, liver, oviduct, gut microbiota

## Abstract

The present study was designed to evaluate the effect of a mixture of Chinese medicinal residues (CMRs) consisting of *Salvia miltiorrhiza* residues (SMR) and *Isatidis Radix* residues (IRR) on productive performance, egg quality, serum lipid and hormone levels, liver and blood antioxidant capacity, oviduct inflammation levels, and gut microbiota in the late-laying stage. A total of 288 fifty-four-week-old *BaShang* long-tailed hens were divided into four groups. The feed trial period was 8 weeks. The control group was fed the basic diet as a CCMR group, supplemented with 3, 4, and 6% for the experimental groups LCMR, MCMR, and HCMR. The egg production rate of the MCMR group was 8.1% higher than that of the CCMR group (*p* < 0.05). Serum triglyceride (TG) levels of hens of the CMR-supplemented group were significantly decreased than those of the CCMR group (*p* < 0.05). The group supplemented with different levels of CMR had significantly higher serum HDL-C levels compared with the control group (*p* < 0.05). Follicle-stimulating hormone (FSH) and luteinizing hormone (LH) levels were remarkably increased for the LCMR and MCMR groups and significantly decreased for the HCMR group compared to CCMR (*p* < 0.05). Serum and liver glutathione peroxidase (GSH-PX) activities were significantly increased, and malondialdehyde (MDA) levels were significantly decreased in the MCMR group compared to the CCMR group (*p* < 0.05). The expression levels of tubal inflammatory factor markers (IL-4, IL-1β, TNF-α) in the MCMR and HCMR groups were consistent with the pathological findings of the sections. As for cecal microbiota, supplementation with CMR affected the alpha diversity of the cecum microbiome at the genus level. The Shannon index was significantly higher in the MCMR group than in the CCMR and HCMR groups (*p* < 0.05). Supplementation with different levels of CMR mainly regulated the ratio of intestinal *Firmicutes* to *Bacteroidetes* and the abundance of phyla such as *Proteobacteria*. In addition, CMR supplementation at different levels in the diet enriched lipid-metabolizing bacteria, such as *Bacteroides* and *Ruminococcus_gnavus_group*. Furthermore, according to linear discriminant analysis (LDA) effect size (LEfSe) analysis, the MCMR group showed an increase in the number of short-chain fatty acid-producing bacteria *Romboutsia* and fiber-degrading specialized bacteria *Monoglobus*. Therefore, supplementation of appropriate amounts of CMR to the diet of laying hens enhanced reproductive hormone levels, hepatic antioxidant capacity, and lipid metabolism, alleviated the levels of oviductal inflammatory factors, and modulated the abundance structure of bacterial flora to improve the late-laying performance and egg quality. The results of the current study showed that CMR is a beneficial feed supplement for chickens when added in moderation.

## 1 Introduction

The policy of reducing and banning the use of antibiotics is closely linked to hen welfare. The problems of drug residues and bacterial resistance caused by the misuse of antibiotics are a serious threat to animal health and food safety. In 2006, the European Union completely banned the use of growth-promoting antimicrobials in poultry feeds (1). According to the Ministry of Rural Affairs Announcement No. 194, in China, all growth-enhancing drug feed additives, except for traditional Chinese medicines, have been banned since 2020. Therefore, research and development of alternatives to antibiotics should be accelerated to meet the needs of the poultry industry and the demand for healthy green egg and poultry products. Chinese medicine resources plant-feeding functional products both to prevent disease and promote growth. The dietary addition of hawthorn leaf flavonoids can alleviate decreased ovarian function and improve hepatic lipid metabolism in older breeders (2). Marigold fortificant is a good colorant for egg yolks (3).

*Salvia miltiorrhiza* (SM) was first documented in “Shen Nong herbal classic,” which can relieve pain and blood stasis. Its main components include polysaccharides, water-soluble phenolic acids, and fat-soluble diterpenoids (4). Modern pharmacophore studies have shown that phenolic acid ingredient salvianolic acid A binds to disease targets sphingomyelin synthase 2 to exert therapeutic effects on hyperlipidemia (5). The liver is protected *in vivo* by the anti-inflammatory and antioxidant effects of SM polysaccharides (6). *Isatidis Radix* (IR) is a representative Chinese drug that is frequently used for heat-clearing and detoxification. Pharmacological studies have confirmed that IR mainly contains alkaloids, sulfur-containing compounds, phenylpropanoids, and other 19 categories of ingredients (7). IR has hypolipidemic pharmacological activity and can effectively inhibit adipocyte differentiation and lipid synthesis (8). IR polysaccharides have anti-inflammatory (9), lipid regulating (10), and immune cell proliferation effects (11). The use of SM and IR combination can function to tonify the liver and nourish blood. SMR and IRR are by-products of the deep processing of medicinal herbs. The deep processing industries such as traditional Chinese medicine and pharmaceuticals, which consume medicinal herbs as raw materials, produce over 70 million tons of by-products each year (12). The water content of CMRs after the extraction of active ingredients can be as high as 70%, and large piles of CMRs will decay rapidly, leading to the pollution of water sources and the emission of irritating gases (13). The herbal residue contains cellulose, hemicellulose, and lignin, as well as many medicinal components such as flavonoids, alkaloids, and organic acids. There is an urgent need to explore ways to utilize CMR resources in a high value-added way, while at the same time alleviating the pressure of environmental pollution. Previous studies have shown that the polysaccharide SMWP-U&E, isolated from SMR, was confirmed in *in vivo* experiments to improve gut morphology and flora, immunity, and antioxidant capacity (14). Residual glucan composition in IRR has been reported to be as high as 39% (15).

The liver is the primary organ for lipid synthesis and catabolism during egg production. Excessive reactive oxygen species (ROS) accumulation in the liver or bloodstream can lead to disorders of lipid metabolism due to elevated levels of fatty acid synthase and impaired synthesis of very low-density lipoprotein (VLDL) (16, 17). The liver interacts directly with the gut microbiota through the portal circulation to influence gut health (18). Intestinal flora composition and its metabolites, such as 5-methoxyindoleacetic acid and indole, remotely regulate hepatic function via the liver-gut axis (19). However, the poultry age and nutrients drive intestinal flora change. Due to the special reproductive structure and performance of laying hens, pathogenic bacteria in the intestine can be transferred to the oviducts through the cloaca to cause inflammation and immune reactions (20). Feedback regulation of hormones will directly affect ovarian follicle development and hen performance.

The *BaShang* long-tailed hen is the only local poultry variety in Hebei Province that has been listed in the “Poultry Genetic Resources in China.” It is physically resistant to rough feeding. However, extremely low egg production rates in the late-phase laying limit the useful cycle of the species. Therefore, this experiment investigated the effects of CMRs, specifically the mixture of SMR and IRR at a ratio of 1:1, supplementation on production performance, blood lipid levels, antioxidant status, reproductive hormone levels, oviductal inflammation levels, and cecum microbiota in the late-laying stage. This study aimed to provide a preliminary assessment of the potential value of CMR as a dietary supplement for layers.

## 2 Materials and methods

### 2.1 Drugs and reagents

The trial materials SMR and IRR were derived from Shineway Pharmaceutical Group Ltd., Shijiazhuang, China. Drying and crushing of single-extracted residue through 100 mesh sieve 1:1 mixing. The assay kits of total superoxide dismutase (T-SOD), GSH-PX, catalase (CAT), MDA, total cholesterol (TC), TG, low-density lipoprotein-cholesterol (LDL-C), and high-density lipoprotein-cholesterol (HDL-C) were purchased from Nanjing Jiancheng Biotechnology Research Institute, Nanjing, China. The assay kits of estrogen (E2), FSH, LH, and progesterone (Prog) were obtained from Shanghai Enzyme-linked Biotechnology Co., Ltd., Shanghai, China. The M5 Universal Plus RNA Mini Kit (MF167-01) and M5 HiPer SYBR Premix Es Taq (MF787-01) were obtained from Mei5 Biotechnology Co., Ltd., Beijing, China. The SweScript All-in-One First-Strand cDNA Synthesis SuperMix for qPCR (G3337-50) was purchased from Wuhan Service Bio Technology Co., Ltd., Wuhan, China.

### 2.2 Animal groups and handling

This experiment was sanctioned by the Animal Conservation and Utilization Committee of Hebei Agricultural University (grant No.2022161). A total of 288 healthy 54-week-old *BaShang* long-tailed hens with similar body weights were obtained from Hebei Green Field Poultry Technology Co., Ltd. (Zhangjiakou, China). Laying hens were kept in indoor cages (3 hens per cage). Each cage contained water bottles and feed buckets. The indoor temperature and humidity were 25°C and 65%, respectively. Furthermore, 16 h of light in the room were maintained, and the room was kept clean and tidy. A total of 72 hens were in each group, with six replicates in each group. Randomly divided into a control group (CCMR), 3% CMR group (LCMR), 4% CMR group (MCMR), and 6% CMR group (HCMR), basal diet supplemented with 3, 4, and 6% CMR, respectively. CMR was added to the trial for 8 weeks. The basal diets were formulated to meet nutrient requirements (Table 1).

**Table 1:** Composition and nutrient levels of the basal diet (%, air-dry basis).

Ingredients/%	
Corn	62.0
Soybean meal	23.0
DL-Methionine	0.22
Gluten meal	2.00
Limestone	6.33
Premix^a^	4.65
Salt	0.30
Dicalcium phosphate	1.50
Total	100

Nutrient levels/%^b^	
Metabolizable energy (MJ/kg)	11.1
Crude protein	15.8
Methionine	0.33
Lysine	0.73
Available phosphorus	0.39
Methionine + Cysteine	0.58
Calcium	3.45
Total phosphorus	0.45

a Provided the following per kilogram of diet: vitamin A, 120000–200,000 IU; vitamin D3, 50,000–100,000 IU; vitamin E, 200 mg; vitamin K3,10–100 mg; vitamin B1, 18 mg; vitamin B2, 60 mg; vitamin B6, 40 mg; calcium pantothenate, 200 mg; nicotinamide, 400 mg; biotin, 1 mg; folic acid, 10 mg; vitamin B12, 0.3 mg; Mn, 1,000–3,000 mg; I, 7–100 mg; Fe, 1,000–15,000 mg; Cu, 120–500 mg; Zn, 1,100–2,400 mg; Se, 3–10 mg; Ca %, 8; P %,1.6.

b Nutrient levels were calculated values.

### 2.3 Sample collection

Eggs are collected and recorded at 3 p.m. daily. At the end of the 8th week, two chickens were taken from each replicate group. Blood samples (5 mL) were collected from each chicken under the wings after 8 h of fasting. These two chickens were then euthanized by jugular bloodletting. Serum samples were separated using a centrifuge at 1,500 × *g* for 10 min at 4°C and stored at −20°C for measurement. Magnum and shell gland of oviducts and cecum contents of (three hens’ ceca contents were mixed into one sample) laying hens were stored in a refrigerator at −80°C for measurement. Oviduct magnum and shell gland tissues were fixed with 4% paraformaldehyde.

### 2.4 Analysis of nutrient and medicinal components in CMR

In the experiment, the SMR and IRR mixture at a ratio of 1:1 was weighed at 3 g. Three parallel replicate samples were set up. Crude protein was assayed using the Kjeldahl method. Crude ash was assayed using the cauterization method. Crude fat was assayed using the Soxhlet extraction method. The analysis of crude fiber, acid detergent fiber, and neutral detergent fiber was done using automatic fiber apparatus (A2000i, ANKOM, America). The content of total flavonoids in CMR was determined using the NaNO2-Al (NO3)3 color developing method (21). For crude polysaccharide, alkaloids, and total saponin test, refer to the total flavonoid test method. Glucose was used as the standard to establish the standard curve. The crude polysaccharide was determined using the phenol-sulfuric acid method at 490 nm with a UV spectrophotometer (UV-6850, JENWAY, United States). Reisner salt can bind to alkaloids in acidic media to form a complex precipitate that forms a purplish-red solution detected at UV 525 nm. Oleanolic acid was used as the standard to establish the standard curve. The samples were added with vanillin-glacial acetic acid solution and perchloric acid, respectively, to determine the absorbance at UV 540 nm and calculate the content of total saponins in CMR.

### 2.5 Production performance and egg quality

During the trial, the number of eggs laid, egg weight, average daily feed intake, dead elimination quantity, and daily feed consumption were recorded for each replicate, and the total egg production rate and feed-to-egg ratio were calculated. At the end of weeks 4 and 8, 12 eggs were taken from each group. Egg weight, Eggshell weight, and yolk weight were weighed using an electronic balance. An egg quality analyzer (EA-01, Orka Food Ltd., Israel) was used to determine albumen height, yolk color, and Haugh unit. Eggshell thickness at the large, middle, and small ends of the egg and the long and short diameter of the egg were determined using electronic digital calipers. The egg shape index and shell index were calculated. Eggshell index = Eggshell weight / 4.68 × (Egg weight)^2/3^ (22). F / E = feed consumption to egg weight ratio.

### 2.6 Serum biochemical and hormone level tests

The serum levels of TC (A111-1-1), TG (A110-1-1), LDL-C (A113-1-1), and HDL-C (A112-1-1) were determined by an enzyme label (DG5033A, East China Electronics Group Medical Equipment Co., Ltd., Nanjing, China) with commercial diagnostic kits. The serum levels of E2 (YJ023194), FSH (YJ042764), LH (YJ611689), and Prog (YJ059935) were determined by an enzyme label (RT-6100, Rayto Life and Analytical Sciences Co., Ltd., China) with commercial diagnostic kits.

### 2.7 Antioxidant indicator tests

The liver tissue was ground with saline in a ratio of 1:10, centrifuged, and the supernatant was taken for testing. Total protein in the supernatant was detected using the Total Protein Assay Kit (Caulmers Brilliant Blue). Measurement of T-SOD (A001-1-2), GSH-PX (A005-1-2), CAT (A007-1-1) activities, and MDA (A003-1-1) contents of serum and liver was determined by enzyme labeling apparatus (DG5033A, East China Electronics Group Medical Equipment Co., Ltd., Nanjing, China) with commercial diagnostic kits.

### 2.8 Histomorphometric analysis

The 4% paraformaldehyde-soaked oviduct magnum and shell gland were taken, fixed, dehydrated using graded alcohol, and soaked in wax. They were paraffin-embedded and sectioned using Leica RM2016; slides were fished and dried, followed by hematoxylin–eosin (HE) staining. Histopathological changes were viewed under a photomicroscope (NIKON ECLIPSE E100, NIKON, Japan).

### 2.9 Real-time polymerase chain reaction analysis

Gene expressions in the oviduct magnum and shell gland (IL-4, IL-1β, TNF-α) were detected using real-time quantitative reverse transcription-polymerase chain reaction (RT-PCR) (BIO-RAD, Hercules, CA). Total RNA was extracted from the magnum and shell gland using M5 Universal Plus RNA Mini Kit, and reverse transcription using the SweScript All-in-One First-strand cDNA Synthesis SuperMix for qPCR Kit. Quantitative real-time PCR was conducted using the M5 HiPer SYBR Premix Es Taq kit. Primer sequences are shown in Table 2. The relative expression of mRNA was determined by the 2^−ΔΔCt^ method.

**Table 2:** Primer sequences.

Target genes	GenBank accession No.	Primers sequences	Product length/bp
GAPDH	NM_204305.2	F: TCCATGCCATCACAGCCACAC R:ATGACTTTCCCCACAGCCTTAGC	130
IL-4	NM_001007079.2	F:GTGCCCACGCTGTGCTTAC R:AGGAAACCTCTCCCTGGATGTC	83
IL-1β	NM_204524.2	F:GGTCAACATCGCCACCTACA R:CATACGAGATGGAAACCAGCAA	86
TNF-ɑ	NM_204267.2	F:GCCCTTCCTGTAACCAGATG R:ACACGACAGCCAAGTCAACG	71

### 2.10 Bacterial DNA extraction and 16S rRNA gene sequencing

Genomic DNA was extracted using the CTAB method. The extracted DNA concentration was measured using the NanoDrop 2000 spectrophotometer (TFS, Waltham, MA, EUA). The extracted DNA is amplified by PCR in the reaction system. Primers 341F and 806R were used for PCR amplification of the V3-V4 region. The PCR products were purified by Qiagen using the gel recovery kit, and the libraries were constructed using the NEBNext^®^ Ultra^™^ IIDNA Kit, and libraries were tested and quantified by Q-PCR using the Agilent 5,400. The sequencing and bioinformatics analysis were performed by Novo Genome Bioinformatics Technology Co., Ltd. (Beijing, China).

Each de-duplicated sequence generated after noise reduction using DADA2 was called ASVs (amplicon sequence variants). Subsequently, the QIIME2 classify-sklearn algorithm was used. Species annotation was performed for each ASV using a pre-trained Naive Bayes classifier. Subjected to diversity analysis (Shannon), a metric-free multidimensional calibration method (NMDS) analysis was performed using the Bray-Curtis method on ASVs to show diversity. LEfSe analyzed inter-group differences (LDA score > 3). The Spearman rank correlation was used to investigate the correlation between serum biochemical and microbial populations.

### 2.11 Statistical analysis

The data were analyzed using IBM SPSS Statistics software (version 25.0, SPSS, Chicago, IL). One-way analysis of variance (ANOVA) and Duncan’s multiple comparison tests were performed between groups. A *p*-value of less than 0.05 (*p* ≤ 0.05) indicates a significant difference among groups.

## 3 Results

### 3.1 Main medicinal components and nutrition in CMRs

The main medicinal and nutritional ingredients in CMRs are shown in Table 3. The content of crude polysaccharide in CMRs was 25.3 mg/kg, followed by a saponin content of 20.8 mg/g, containing small amounts of flavonoids and alkaloids of 13.1 mg/g and 12.0 mg/g, respectively. The nutritional analysis of CMRs revealed a crude fiber content of 25 mg/g and lower crude protein and crude fat content. The disadvantages of using it as animal feed are poor palatability, low digestibility, and low nutritional value.

**Table 3:** Main medicinal components and nutrition in CMRs.

	Content (mg/g)
Nutritional composition	
Dry matter	86.0
Crude ash	7.17
Crude protein	12.6
Crude fat	13.5
Crude fiber	25.0
Neutral detergent fiber	57.5
Acid detergent fiber	31.8
Medicinal ingredients	
Crude polysaccharide	25.3
Total flavonoids	13.1
Total alkaloids	12.0
Total saponins	20.8

### 3.2 Effect of CMR supplementation in the diet on production performance and egg quality

In the fourth week, yolk weight was significantly increased (*p* < 0.05) in the MCMR and HCMR groups compared with the CCMR group, and there was no significant difference between the CMR groups (*p* > 0.05; Table 4). Differences in ADFI levels between groups were significant and were significantly quadratic with changes in CMR supplementation levels (*p* < 0.05). In the eighth week, the MCMR group had a significantly lower (*p* < 0.05) feed-to-egg ratio, but the egg production rate of the MCMR group was 8.1% higher than that of the CCMR group and 8.2% higher than that of the HCMR group (*p* < 0.05). The eggshell index of the HCMR group was significantly lower than that of the other groups (*p* < 0.05).

**Table 4:** Effect of CMR supplementation in diets on production performance and egg quality.

Items	CMR Level, g/kg of feed				Pooled SEM	*p*-value		
	CCMR	LCMR	MCMR	HCMR		ANOVA	Linear	Quadratic
4 week								
Laying rate/%	40.0	45.1	42.9	41.5	1.09	0.481	0.844	0.208
Egg weight	57.8	58.9	59.4	57.3	0.33	0.093	0.699	0.018
ADFI (g/d)	93.6^a^	88.6^b^	87.9^b^	93.4^a^	0.72	<0.001	0.573	<0.001
F/E	4.05	3.38	3.44	3.94	0.12	0.067	0.753	0.012
Shape index	1.29	1.33	1.32	1.30	0.01	0.230	1.000	0.048
Albumen height (mm)	9.13	8.52	8.90	7.65	0.29	0.298	0.125	0.602
Haugh unit	86.8	93.3	95.7	92.1	1.30	0.086	0.096	0.046
Yolk color	8.83	8.33	8.33	9.67	0.22	0.087	0.174	0.031
Yolk weight (g)	16.7^b^	18.2^ab^	19.4^a^	19.0^a^	0.34	0.012	0.003	0.116
Eggshell index	7.96	7.96	8.65	8.45	0.15	0.076	0.038	0.683
8 week								
Laying rate/%	38.2^b^	43.9^ab^	46.3^a^	38.1^b^	1.39	0.049	0.897	0.013
Egg weight	60.3	59.6	60.0	57.9	0.36	0.067	0.029	0.283
ADFI (g/d)	95.8	98.1	97.2	96.2	0.74	0.729	0.983	0.316
F/E	4.17^ab^	3.75^bc^	3.50^c^	4.36^a^	0.12	0.006	0.603	0.001
Shape index	1.36	1.33	1.36	1.34	0.01	0.580	0.904	0.903
Albumen height (mm)	8.31	8.47	8.55	7.88	0.10	0.072	0.158	0.033
Haugh unit	80.3	86.1	87.3	83.2	1.37	0.272	0.408	0.075
Yolk color	7.92	8.17	8.92	9.25	0.25	0.188	0.034	0.932
Yolk weight (g)	17.8	17.4	17.5	17.3	0.27	0.951	0.670	0.826
Eggshell index	8.06^a^	8.09^a^	8.13^a^	7.31^b^	0.12	0.047	0.039	0.074

F, feed consumption; E, egg weight ratio, F/ E, feed consumption to Egg weight ratio; ADFI, average daily feed intake; CMR, the mixture of SMR and IRR at a ratio of 1:1; CCMR, basal diet; LCMR, 30 g/kg CMR supplementation in the basal diet; MCMR, 40 g/kg CMR supplementation in the basal diet; HCMR, 60 g/kg CMR supplementation in the basal diet; SEM, standard error of the mean; The same index without the same superscript a–c values significantly (*p* < 0.05).

### 3.3 Effects of CMR supplementation in the diet on serum biochemical and hormonal parameters

**Figure 1:**
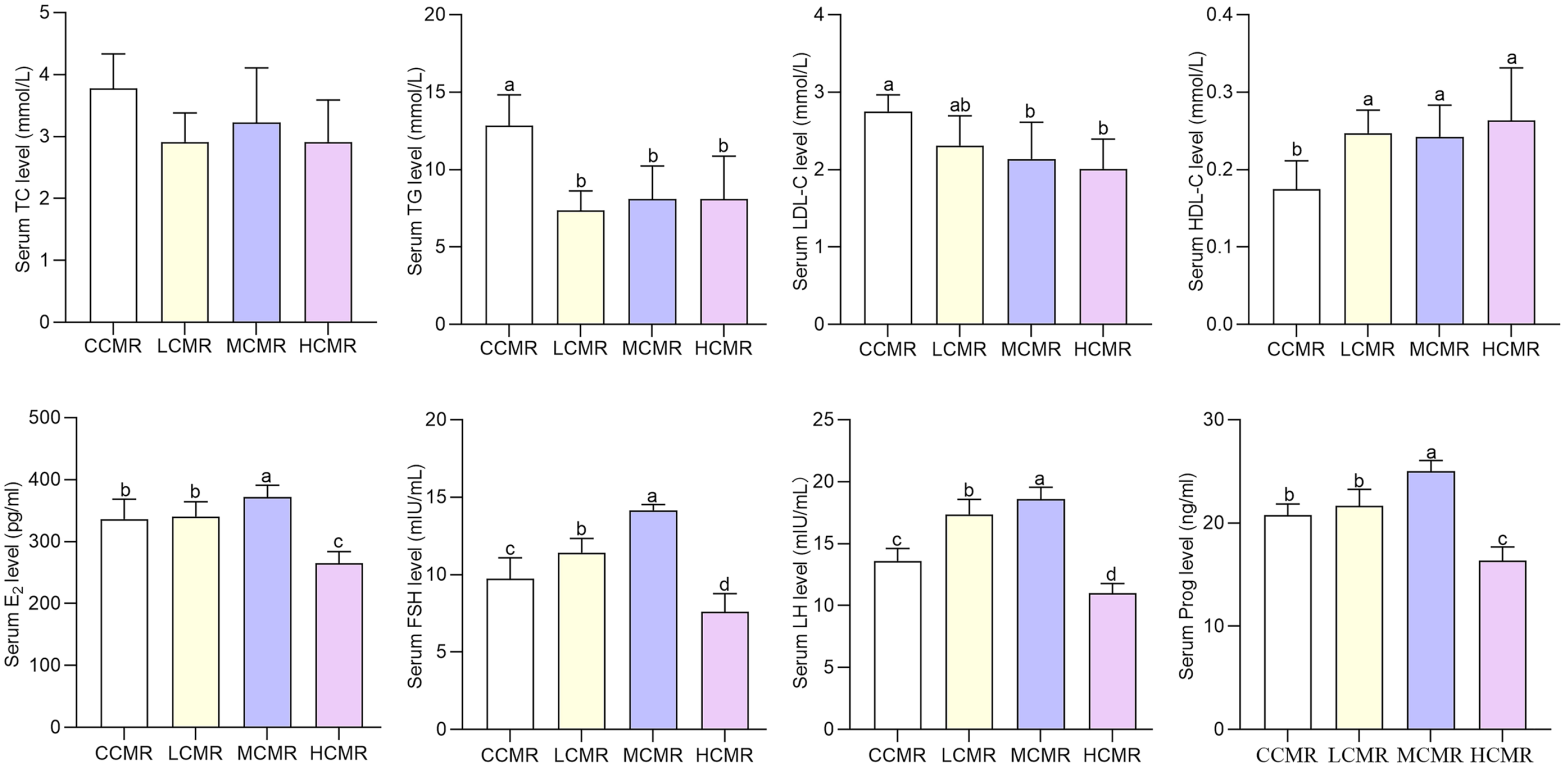
Effect of CMR supplementation in diets on serum biochemical and hormone parameters. CCMR (basal diet), LCMR (30 g/kg CMR supplementation in the basal diet), MCMR (40 g/kg CMR supplementation in the basal diet), and HCMR (60 g/kg CMR supplementation in the basal diet). The same index without the same superscript a-d value significantly (*p* < 0.05).

The serum levels of TG were significantly lower, and HDL-C levels were significantly higher in laying hens after CMR supplementation in the diets compared with the CCMR group (*p* < 0.05; Figure 1). The serum LDL-C levels were significantly lower in the MCMR and HCMR groups than in the CCMR group (*p* < 0.05), but there was an insignificant difference among the three supplemental CMR groups (*p* > 0.05).

Serum levels of FSH and LH were significantly higher in the LCMR and MCMR groups than in the CCMR group (*p <* 0.05, Figure 1). The serum levels of Prog and E2 in laying hens in the MCMR group were significantly higher, whereas the serum levels of E2 and Prog in laying hens in the HCMR group were significantly lower than those in the CCMR group (*p* < 0.05). Serum FSH and LH levels of laying hens in the MCMR group were significantly higher than those in the LCMR group (*p* < 0.05).

### 3.4 Effect of dietary CMR supplementation on histomorphology

**Figure 2:**
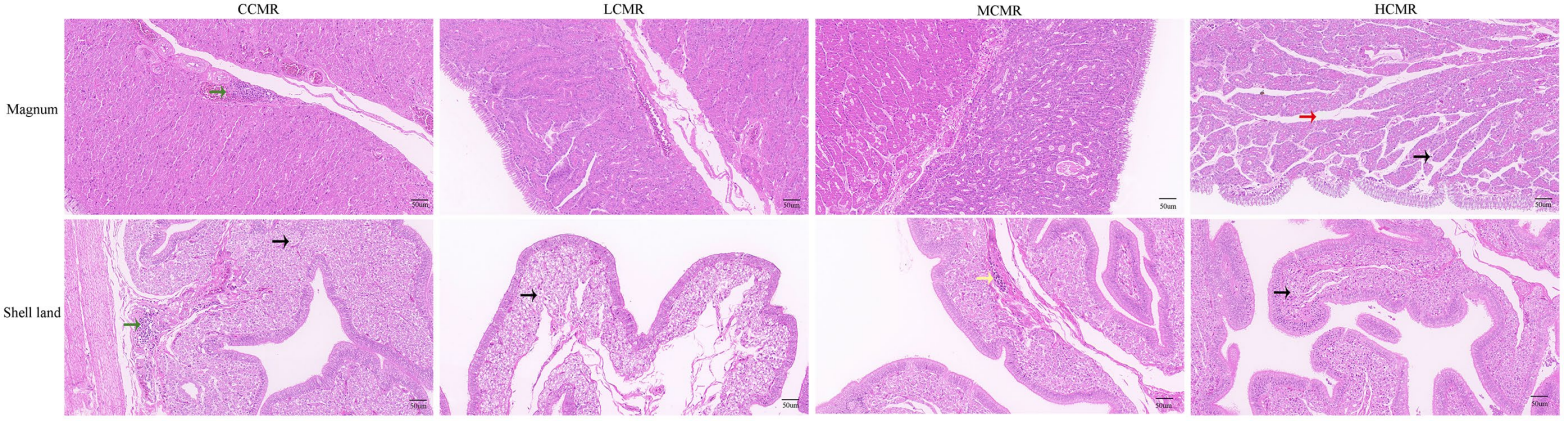
Histomorphometric analysis of the oviduct magnum and shell gland. The black arrows in the figure indicate cytoplasm showing vacuolation; the red arrows indicate secondary folds; the green arrows indicate foci of lymphocytic inflammatory infiltrate; and the yellow arrows indicate leukocytes.

The results of the impact of CMR addition to the diet on the oviductal histomorphology of hens are shown in Figure 2. A small amount of focal infiltration of lymphocytes can be seen in the oviduct magnum submucosa of the CCMR group. In the HCMR group, there were many secondary folds of cilia in the magnum mucosa layer, and there were severe wrinkles. The columnar ciliated cell layer in the CMR-supplemented group was thickened to varying degrees. There is an occasional increase in white blood cell count in the oviduct shell gland of the MCMR group. In the other three groups, the cytoplasm of the gland cells in the shell gland is vacuolated.

### 3.5 Effect of dietary CMR supplementation on antioxidation indices

The effect of CMR on serum and liver antioxidant capacity is shown in Table 5. There were insignificant differences in serum and liver T-SOD activity and CAT activity between the groups (*p* > 0.05). The activity of GSH-PX in the MCMR group was significantly higher, and MDA content was significantly lower compared with the CCMR group (*p* < 0.05). There was an insignificant difference in GSH-PX activity and MDA content among the CMR-supplemented groups (*p* > 0.05). Significant secondary changes in serum and hepatic GSH-PX activity, as well as MDA content, were observed with increasing levels of the CMR additive (*p* < 0.05).

**Table 5:** Effect of dietary CMR supplementation on serum and liver antioxidant capacity.

Items	CMR Level, g/kg of feed				Pooled SEM	*p*-value		
	CCMR	LCMR	MCMR	HCMR		ANOVA	Linear	Quadratic
Serum								
T-SOD (U/mL)	495.53	522.98	544.68	466.98	12.5	0.136	0.544	0.037
GSH-PX (U/mL)	2601.25^b^	3016.18^ab^	3226.08^a^	3003.01^ab^	84.4	0.050	0.045	0.043
CAT (U/mL)	1.31	1.24	1.21	0.82	0.07	0.074	0.022	0.247
MDA (nmol/mL)	2.54^a^	1.49^b^	1.53^b^	1.87^b^	0.13	0.005	0.037	0.002
Liver								
T-SOD (U/mgprot)	1417.28	1460.35	1509.15	1426.57	16.5	0.192	0.593	0.060
GSH-PX (U/mgprot)	80.77^b^	96.50^a^	96.73^a^	92.48^ab^	2.39	0.045	0.071	0.026
CAT (U/mgprot)	25.7	26.4	32.0	24.9	1.18	0.131	0.742	0.097
MDA (nmol/mgprot)	0.66^a^	0.51^b^	0.51^b^	0.62^ab^	0.02	0.048	0.504	0.008

T-SOD, superoxide dismutase; GSH-PX, glutathione peroxidase; CAT, catalase; MDA, malondialdehyde. CMR, the mixture of SMR and IRR at a ratio of 1:1; CCMR, basal diet; LCMR, 30 g/kg CMR supplementation in the basal diet; MCMR, 40 g/kg CMR supplementation in the basal diet; HCMR, 60 g/kg CMR supplementation in the basal diet; SEM, standard error of the mean; The same index without the same superscript a and b values significantly (*p* < 0.05).

### 3.6 Effect of dietary CMR supplementation on oviduct-related mRNA gene expression

**Figure 3:**
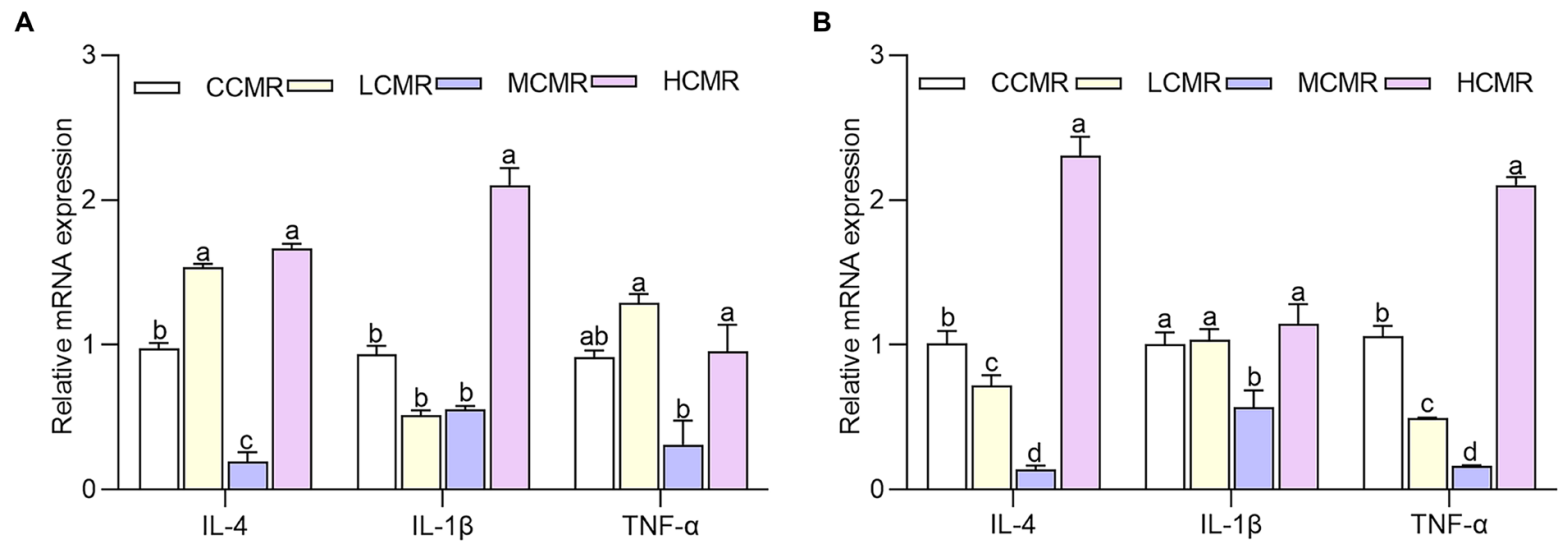
Inflammatory factor gene expression in the oviduct. **(A)** Oviduct magnum. **(B)** Oviduct shell gland. The same index without the same superscript a–d values significantly (*p* < 0.05).

Inflammatory factor IL-4 expression levels in the oviduct magnum were significantly decreased after supplementation of MCMR in the diets compared with those in the CCMR group, whereas IL-1β expression levels were significantly increased in the HCMR group (*p* < 0.05; Figure 3A). The level of TNF-ɑ expression was lowest in the MCMR group compared with the CMR-supplemented group (*p* < 0.05). The difference in the expression level of inflammatory factors IL-4 and TNF-α in the oviduct shell gland was significant among groups (*p* < 0.05; Figure 3B).

### 3.7 Effect of dietary CMR supplementation on cecal microbiota composition

**Figure 4:**
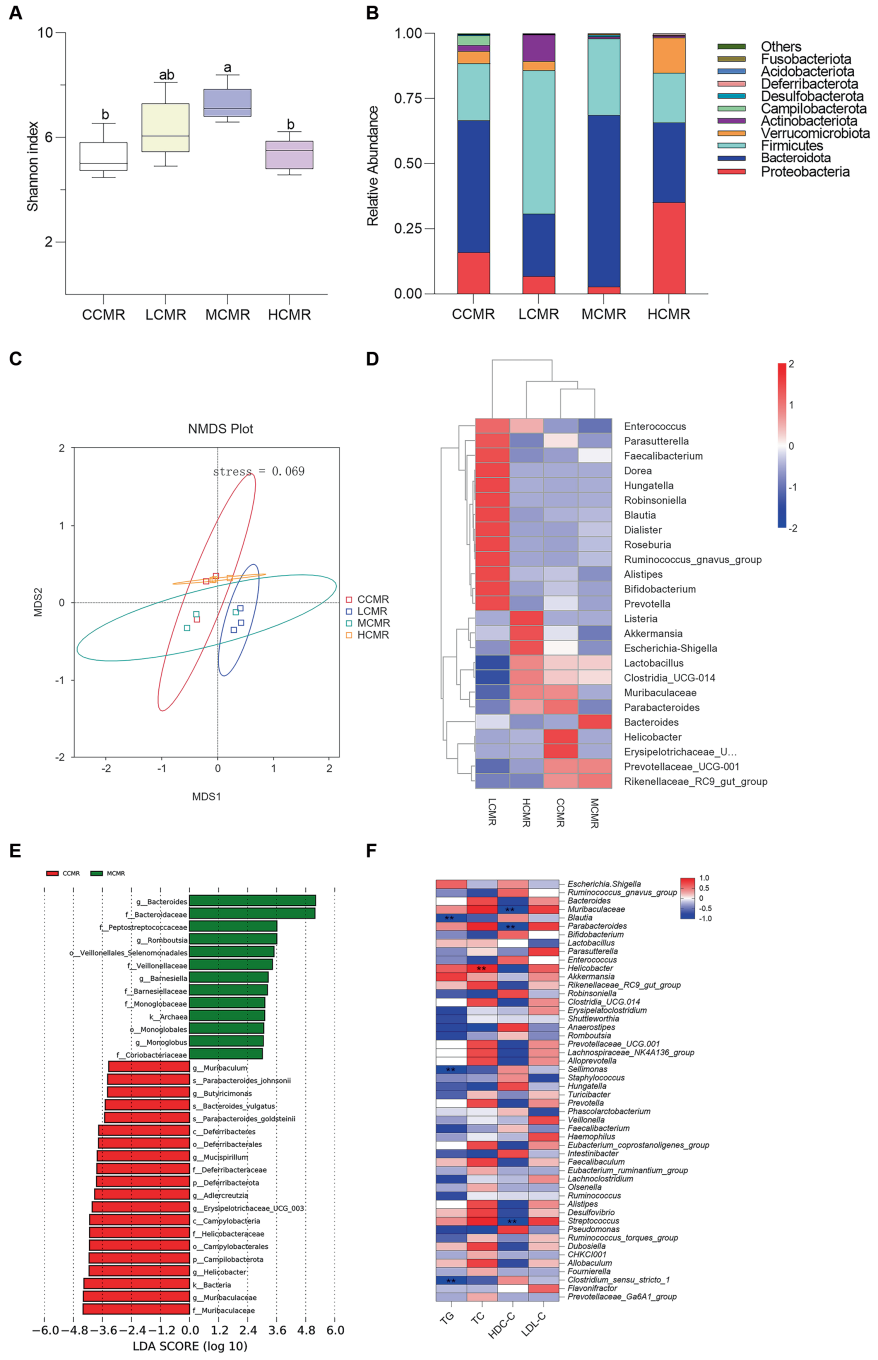
Analysis of cecum gut microbiota. **(A)** The Shannon index; **(B)** phylum level relative abundance; **(C)** NMDS analysis; **(D)** genus-level species abundance clustering heat map; **(E)**. LEfSe analysis (LDA score > 3); and **(F)** Spearman analysis at the genus level between serum biochemical indices and cecal microbiota (Three hens’ ceca contents were mixed into one sample).

In the microbial analysis, an average of 74,410 filtered high-quality sequences were obtained. The alpha diversity analysis using the Shannon index showed that the MCMR group had a significantly higher diversity than the control and HCMR groups (*p* < 0.05, Figure 4A). The stress values were less than 0.2 (*p* = 0.069; Figure 4C) for each group indicating the feasibility of using NMDS to accurately respond to different levels in different samples. The dominant flora were Firmicutes and Bacteroidetes followed by *Verrucomicrobiota* and *Proteobacteria* at the phylum level (Figure 4B). Enrichment abundance varied considerably between groups at the genus level, with the MCMR group dominated by *Bacteroidetes, Rikenellaceae_RC9_gut_group*, and *Lactobacillus* (Figure 4D). LEfSe analysis of bacteria as biomarkers differentiated the microbiota of the control and MCMR groups. The MCMR group was enriched with the organic matter-degrading bacteria *Romboutsia*, *Veillonellaceae*, *Barnesiella*, and *Monoglobus* (Figure 4E). The correlation between cecum microbiota genus levels and serum biochemical indices was analyzed using the Spearman analysis, and the results are shown in Figure 4F. *Blautia*, *Sellimonas,* and *Clostridium_sensu_stricto_1* were negatively correlated with serum lipid metabolism (TC, TG, and LDL-C). *Muribaculaceae*, *Parabacteroides*, *Helicobacter, and streptococcus* had a positive correlation with serum lipid metabolism.

## 4 Discussion

The active ingredients are still present in the residue of Chinese medicines after decoction or extraction. For example, the HPLC analysis of the residue after *Sophora flavescens* water extraction by ethyl acetate ultrasonic re-extraction revealed that the main component contained various flavonoids (kurarinone and sophoraflavanone G) (23). Our study revealed that polysaccharides, flavonoids, alkaloids, and saponins were detected in CMR. Modern pharmacology has proved that its constituents have anti-inflammatory, antioxidant, hepatoprotective, and other biological activities (24, 25). Furthermore, CMRs contain some basic nutrients. Therefore, CMRs have the conditions to be developed into feed supplements.

Production performance is normally used to assess the condition of the laying hens’ bodies. Our study showed that supplementation of MCMR in the diet increased egg production and reduced the feed-to-egg ratio. The increased egg production and reduced feed-to-egg ratio may be due to improved hormone levels and an increased abundance of beneficial bacteria that degrade carbohydrates. The hypothalamic–pituitary (FSH and LH)-gonadal (E2) axis regulates follicular development and productive performance. The reproductive hormone FSH functions to promote follicular growth, development, and maturation, and LH induces follicular ovulation. Levels of serum FSH and LH were significantly higher in the LCMR and MCMR groups compared to the group without added CMR. Serum E2 and Prog levels were also significantly increased in the MCMR group compared to the other groups. Therefore, supplementation with CMR enhances the synergistic effect of secreted hormones, thereby promoting production performance in laying hens. The high content of crude fiber and lignin in the diet reduces palatability, and anti-nutritional factors such as tannins and phytic acid reduce the apparent digestibility of nutrients. Small mammals that consume high-fiber foods, in addition to increasing food intake to compensate for decreased digestibility, additionally reduce basal metabolic rate and non-shivering thermogenesis levels to compensate for reduced energy acquisition efficiency by reducing energy expenditure (26, 27). There was evidence that reduced egg production was directly correlated with reproductive hormones and liver metabolism (28, 29). The mean daily feed intake at 1–4 weeks and egg production rate at 4–8 weeks in the HCMR group were insignificantly different from those of the control group. Meanwhile, the disturbed intestinal flora of the HCMR group may affect the brain-intestinal-hepatic-ovarian axis, leading to a significant decrease in reproductive hormone levels and affecting egg production, and the specific mechanism is subject to further study.

The expression of anti-inflammatory and pro-inflammatory factors produced by the mucosal microwell environment of the oviduct of the laying hen is in dynamic balance under healthy conditions. The process of Gram-negative bacterial death or multiplication releases lipopolysaccharides (LPS), leading to elevated levels of pro-inflammatory factors. IL-4 cytokines are mainly secreted by a subpopulation of Th2 T cells and promote immune responses by binding specifically to interleukin-4 receptors on the surface of target cells (30). Other studies have shown that IL-4 and TNF-α have a dual role in resisting Gram-negative bacterial infections in the organism (31). The expression of anti-inflammatory factor IL-4 and pro-inflammatory factors TNF-ɑ and IL-1β genes was reduced in the MCMR group, probably because the levels of anti-inflammatory factors were correspondingly lowered by the organism to maintain relative homeostasis. The pro-inflammatory cytokine IL-1β affects eggshell ultrastructure through inhibition of calcium-binding protein expression and ca^2+^ transport in the endometrium (32). The HE staining results corroborate the molecular results that CMR can reduce inflammation in the fallopian tube tissue. The eggshell index refers to the eggshell weight per unit area. *Escherichia-Shigella* has been reported to be associated with eggshell quality in eggs (33). Due to the special reproductive structure of laying hens, the oviduct increases exposure to harmful bacteria, so abnormal calcium synthesis in the oviduct shell gland and impaired immune function caused a decrease in the eggshell index in the HCMR group.

After the high-intensity metabolic phase of the peak laying period in laying hens, many diseases of the body are caused by the imbalance of the redox system disrupted by reactive oxygen species. Enzymatic antioxidant defense systems include T-SOD, GSH-PX, and CAT. MDA is a peroxidation product of lipids during oxidative stress. It has been shown that the addition of quercetin and daidzein, a natural antioxidant, significantly increased antioxidant enzyme levels and decreased the content of MDA in laying hens (34). The same results were found in our study, where the serum and liver of CMR-supplemented laying hens showed different increases in GSH-PX activity and decreases in MDA content compared to the normal diet group. The metabolic disorders of hepatic lipids in the late egg-laying period directly affect the production of yolk precursor substances (28). Our study showed a decrease in the serum LDL-C/HDL-C ratio and a significant decline in serum TG levels in the CMR-supplemented group, suggesting an improvement in hepatic lipid metabolism levels in laying hens. *Bifidobacterium* and *Lactobacillus* are beneficial bacteria for lipid metabolism (35). The LCMR group was enriched with *Bifidobacterium*, while *Lactobacillus* was enriched in both the HCMR and MCMR groups with a higher abundance than the control group. Prior studies have confirmed the role of *Parabacteroides* in regulating lipid metabolism through transforming bile acid and succinic acid (36). The abundance of *Parabacteroides* in our study was low in the LCMR and MCMR groups but high in the HCMR and CCMR groups. However, lipid levels remained low for the HCMR group, which may be related to *Akkermansia*. Oral *Akkermansia* has been reported to reverse metabolic abnormalities caused by a high-fat diet (37).

Gut microflora are important in the maintenance of intestinal physiological functions and related activities. Insoluble fiber plant feed expands when it encounters water. This expansion increases the contact area between surimi and the epithelial cells of intestinal villi, prolongs the retention time and retention rate of surimi in the gastrointestinal tract, and accelerates the reproduction of harmful bacteria. This can potentially lead to intestinal diseases and reduce the feed conversion rate (38, 39). The relative abundance ratio of *Bacteroidetes* to *Firmicutes* increased during the late egg-laying period, while the abundance of *Proteobacteria* decreased (40). The results of this trial are consistent with previous ones, and we found that the LCMR and MCMR groups increased the diversity of the cecum microflora, dominated by *Firmicutes* and *Bacteroidota*. However, over-supplementation decreased the diversity of the flora, dominated by *Proteobacteria* and *Bacteroidota*. *Proteobacteria* include some conditionally pathogenic bacteria, such as *Salmonella* and *E. coli*. Similarly, the HCMR group enriched for *Listeria* and *Escherichia-Shigella* may be a causative agent unfavorable to the organism. Previous studies suggest *Escherichia-Shigella* may contribute to hepatic inflammation with fibrosis in NAFLD pathogenesis (41).

Cluster analysis of the intestinal genus level. The LCMR group was enriched with the *Ruminococcus_gnavus_group*, which has been shown to correlate with serum TC levels (42). The MCMR group was enriched with *Bacteroides* and *Rikenellaceae_RC9_gut_group*. Changes in the relative abundance of Bacteroides may alter body glycolipid metabolism (43). However, in our study, *Rikenellaceae_RC9_gut_group* was a mixed-review bacterial genus that was positively correlated with serum TC and TG levels, or possibly with host species. LEfSe analysis for the control and MCMR groups revealed that the MCMR group was enriched with the short-chain fatty acid-producing bacterium *Romboutsia* (44). It has been shown that *Barnesiella* is associated with the regulation of bile acids (45). Alterations in gut microbiology mediated by abnormal bile acid secretion are influenced by a high-fat diet (46). *Monoglobus* is a specialized bacterium that breaks down pectin, the main non-cellulosic component of plant cell wall polysaccharides (47, 48). Monoglobus promotes the production of short-chain fatty acids and improves the intestinal microenvironment (49). Gut microbes and their metabolites are hubs connecting the liver-brain-reproductive signaling axis to nutritional strategies that regulate laying hen performance, health, and welfare.

## 5 Conclusion

In summary, the supplementation of 40 g/kg CMR in the hen diet improved antioxidant capacity, reduced the blood lipid level, alleviated the inflammation of oviducts, and regulated the flora structure. Thus, CMRs alleviated the state of health in late-phase laying hens to improve performance. Note that over-addition of CMRs can have adverse effects on laying hens, and the exact mechanism needs to be studied in depth. Moderate amounts of SMR and IRR have the potential to be developed as feed supplements for roughage-tolerant poultry.

## Data availability statement

The original contributions presented in the study are publicly available. This data can be found at: https://www.ncbi.nlm.nih.gov/bioproject/, PRJNA1060451.

## Ethics statement

The animal studies were following approval of study protocols by the Animal Protection and Utiliza-tion Committee of the Hebei Agricultural University. The studies were conducted in accordance with the local legislation and institutional requirements. Written informed consent was obtained from the owners for the participation of their animals in this study.

## Author contributions

ZL: Data curation, Methodology, Software, Writing – original draft, Writing – review & editing. NM: Data curation, Software, Writing – original draft, Writing – review & editing. XG: Methodology, Supervision, Writing – review & editing. WS: Project administration, Supervision, Writing – review & editing. XM: Conceptualization, Writing – review & editing. JY: Data curation, Writing – review & editing. ZZ: Conceptualization, Writing – review & editing. JL: Funding acquisition, Supervision, Writing – review & editing.
